
JOURNAL INFORMATION
==============================
NLM Title Abbreviation: Medizinrecht
Journal ID: Medizinrecht
NLM Journal ID: 8309354

Medizinrecht

ISSN: 0723-8886
EISSN: 1433-8629

ARTICLE INFORMATION
==============================
PMCID: PMC9574160
PMID: 36274781
DOI: 10.1007/s00350-022-6325-4
Article ID: 6325
Article version: 1
Subjects: Aufsätze

Triage und Priorisierung

Brettel Hauke 1
Schuhr Jan C. 2
1 grid.5802.f 0000 0001 1941 7111 Lehrstuhl für Kriminologie, Strafrecht und Medizinrecht, einschließlich Jugendstraf- und Strafvollzugsrecht, Johannes Gutenberg Universität Mainz, Mainz, Deutschland
2 grid.7700.0 0000 0001 2190 4373 Lehrstuhl für Strafrecht und Strafprozessrecht, Medizinrecht und Rechtsphilosophie, Universität Heidelberg, Heidelberg, Deutschland
Electronic publication date: 2022 Oct 17
Print publication date: 2022
Volume: 40
Issue: 10
First page: 801
Last page: 808
Copyright: © Der/die Autor(en) 2022
License: Open Access Dieser Artikel wird unter der Creative Commons Namensnennung 4.0 International Lizenz veröffentlicht, welche die Nutzung, Vervielfältigung, Bearbeitung, Verbreitung und Wiedergabe in jeglichem Medium und Format erlaubt, sofern Sie den/die ursprünglichen Autor(en) und die Quelle ordnungsgemäß nennen, einen Link zur Creative Commons Lizenz beifügen und angeben, ob Änderungen vorgenommen wurden.
Die in diesem Artikel enthaltenen Bilder und sonstiges Drittmaterial unterliegen ebenfalls der genannten Creative Commons Lizenz, sofern sich aus der Abbildungslegende nichts anderes ergibt. Sofern das betreffende Material nicht unter der genannten Creative Commons Lizenz steht und die betreffende Handlung nicht nach gesetzlichen Vorschriften erlaubt ist, ist für die oben aufgeführten Weiterverwendungen des Materials die Einwilligung des jeweiligen Rechteinhabers einzuholen.
Weitere Details zur Lizenz entnehmen Sie bitte der Lizenzinformation auf http://creativecommons.org/licenses/by/4.0/deed.de.
License URL: https://creativecommons.org/licenses/by/4.0/

issue-copyright-statement: © Springer-Verlag GmbH Deutschland, ein Teil von Springer Nature 2022

==============================

**Hauke Brettel und Jan C. Schuhr**

### I. Einleitung

Wahrscheinlich gibt es im Moment kaum ein Thema, auf das hinzuführen so weniger Worte bedarf – wird doch über die Triage nun schon seit mehr als zwei Jahren in der (Fach-)Öffentlichkeit intensiv diskutiert. Die jetzigen Vorbemerkungen halten sich deshalb auch kurz und bestimmen vor allem – zur Schaffung einer gemeinsamen Ausgangslage – den Begriff der Triage, der den nachfolgenden Überlegungen zugrunde liegt:

Das BVerfG hat im Dezember über Vorkehrungen zum Schutz behinderter Menschen für den Fall einer pandemiebedingt auftretenden Triage entschieden. Darin spricht es vom „Fall der Triage“ als Entscheidung über die Zuteilung medizinischer Ressourcen, die nicht für alle Behandlungsbedürftigen ausreichen können[1]. Es geht also um einen Fall der Ressourcenknappheit, um eine Mangelsituation – in der Entscheidung des BVerfG vor allem um den Mangel

Prof. Dr. iur. Dr. med. Hauke Brettel, Inhaber des Lehrstuhls für Kriminologie, Strafrecht und Medizinrecht, einschließlich Jugendstraf- und Strafvollzugsrecht,
Johannes Gutenberg Universität Mainz, Deutschland

Prof. Dr. iur. Jan C. Schuhr, Inhaber des Lehrstuhls für Strafrecht und Strafprozessrecht, Medizinrecht und Rechtsphilosophie, Universität Heidelberg, Deutschland

Der Text beruht auf den Vorträgen der Medizinrechtslehrertagung von Hauke Brettel zu den Teilen I. und III., von Jan Schuhr zum Teil II

*) Schriftliche Fassung des im Rahmen der Tagung der deutschsprachigen Medizinrechtslehrerinnen und Medizinrechtslehrer „Medizinrecht zwischen Unter- und Überregulierung“ vom 13. und 14. 5. 2022 in München gehaltenen Vortrags.
1) BVerfG, Beschl. v. 16. 12. 2021 – 1 BvR 1541/20 –, Rdnr. 1.

an Beatmungsgeräten wegen einer zu großen Zahl von beatmungspflichtigen COVID-Patienten. In einer solchen Mangelsituation gibt es typischerweise nicht nur Kapazitätsengpässe bei den medizinischen Ressourcen, sondern beispielsweise auch bei den zeitlichen oder den psychischen Kapazitäten der Beteiligten.

Die Zuteilung von Beatmungsgeräten stand auch vor der genannten Entscheidung des BVerfG im Zentrum der Diskussion über die „Triage", die sich einzelnen Konstellationen der Auswahlentscheidung bei Ressourcenknappheit mit unterschiedlicher Aufmerksamkeit zuwandte. Auch die Einteilung in Impfklassen beispielsweise war bereits das Ergebnis einer Entscheidung über die Zuweisung der vorhandenen und nicht ausreichenden Ressourcen. Darüber wurde allerdings nicht mit der gleichen Intensität und Leidenschaft diskutiert wie über die Frage nach der Zuteilung von Beatmungsplätzen bzw. von Intensivbetten.

Stets sind brisante Rechtsfragen im Spiel, wenn über die Zuweisung von Ressourcen entschieden werden muss, die den Bedarf nicht decken. Denn in dieser Situation ist nicht zu vermeiden, dass anerkannte Rechtspositionen zurückstehen. Gut nachvollziehbar spricht das BVerfG deshalb von der Triage als einer „extremen Entscheidungssituation"[2], die Fundamente unserer Werteordnung berührt[3]. Die Vorenthaltung von notwendiger medizinischer Hilfe etwa übergeht stets fundamentale Rechtspositionen wie beispielsweise den Rechtsanspruch auf eine notwendige Krankenbehandlung[4] oder das Recht auf Leben[5].

Zugleich ist darin keineswegs ein neuartiges Phänomen zu sehen. Zwar hat das Thema der begrenzten Kapazitäten im Rahmen der Coronavirus-Pandemie besondere Aufmerksamkeit erlangt[6]. Kapazitätsmängel sind jedoch allgegenwärtig, permanent müssen Priorisierungsentscheidungen getroffen werden. Im Gesundheitswesen beispielsweise haben, so paradox es vielleicht auch klingen mag, nicht zuletzt die Fortschritte in der Medizin zu Kapazitätsengpässen beigetragen, indem sie das Spektrum der Behandlungsmöglichkeiten radikal vergrößerten. Im 18. Jahrhundert konnten noch keine Organe transplantiert werden, weshalb man sich über Prioritäten dabei auch keine Gedanken machen musste. Inzwischen aber ist so Vieles möglich, dass längst auch die Grenze des finanziell Machbaren ins Blickfeld gerückt ist – nicht zuletzt, weil viele Behandlungsmöglichkeiten einen Kraftakt der Hochleistungsmedizin darstellen, den man genausowenig wie eine Luxuslimousine der gesamten Weltbevölkerung zur Verfügung stellen kann. Auch deshalb ist das moderne Gesundheitswesen „unersättlich" und der Abstand zwischen dem medizinisch Möglichen und dem ökonomisch Tragbaren von Tag zu Tag größer[7]. Zu Recht bezeichnet die Bundesärztekammer Priorisierungsentscheidungen als zentralen Bestandteil ärztlicher Tätigkeit[8] – und zwar auch dann, wenn es nicht um finanziell Machbares, sondern um Leben und Tod geht. Dabei gibt es nicht nur in der Medizin die Notwendigkeit von Priorisierungen, die Menschenleben kosten können – in der Seenotrettung oder der Bergrettung nach einem Lawinenabgang beispielsweise ist dies auch so.

Schon diese wenigen Beispiele deuten darauf hin, dass die Frage nach dem Lebens- und Rechtsgüterschutz bei Ressourcenknappheit in eine heterogene Fallgruppe hinführt. Unterschiede, zum Beispiel zwischen einer Triage in Kriegssituationen, einem Katastrophenfall oder einer Pandemie, spielen auch in der rechtlichen Diskussion eine Rolle. Im Krieg etwa dient Triage dem Erhalt der Verteidigungsfähigkeit und die verletzten Soldaten befinden sich in der gleichen – von COVID-Patienten abweichenden – Aufopferungssituation.

Trotz aller Unterschiede stellt sich allerdings stets die gleiche Kernfrage der Verteilungsgerechtigkeit: Wie sind von Rechts wegen Ressourcen zu verteilen, die nicht für alle Bedürftigen ausreichen? Vor allem damit beschäftigen sich die nachfolgenden Überlegungen. Es geht um das allgemeine Problem der Kapazitätsengpässe, das weder ein Novum noch ein Spezifikum der COVID-19-Pandemie ist. Dabei meinen wir, dass es im Hinblick auf Gemeinsamkeiten auch einheitliche Umgangsregeln geben muss. Sie sind natürlich besonders brisant, wenn es um Leben und Tod geht – wie eben in einer Pandemie, die auch deshalb zu einer Art von „kollektivem Stresstest" werden kann.

Konkret soll nach dieser Einleitung der Versuch einer Systematisierung (II.) unternommen werden. Dabei geht es im Kern um die Identifikation von Grundentscheidungen der Rechtsordnung, die etwas über das Vorgehen bei der Herleitung von Priorisierungskriterien aussagen. Der Modus der Generierung von Kriterien, nicht die Kriterien selbst, stehen hier im Zentrum der Überlegungen, die sich als „rechtliche" Betrachtung verstehen. Hauptorientierung ist also die Rechtsordnung in der Hoffnung auf rechtliche Antworten. Vor allem sollen mit der Priorisierung verbundene Fragen in eine gedankliche Reihenfolge gebracht und in der Dogmatik verortet werden. Der dann folgende Abschnitt (III.) widmet sich mit erneutem Bezug auf Priorisierungskriterien beispielhaften Konkretisierungen der zuvor hergeleiteten Ergebnisse, bevor ein Schlussteil einen zusammenfassenden Überblick über die Kernthesen gibt.

Damit liegt auch diesem Text eine Priorisierungsentscheidung zugrunde. Denn natürlich reicht der jetzige Darstellungsraum nicht im Ansatz dafür aus, das Thema „Triage und Priorisierung" vollständig abzuhandeln. Nicht einmal der aktuelle Diskussionsstand könnte auf diesem Raum detailliert wiedergegeben werden, vielmehr ist eine Beschränkung auf Einzelaspekte – wie etwa die ex-ante- und ex-post-Triage oder der Lebensschutz – geboten. Denn ein Kapazitätsengpass zwingt auch hier dazu, wichtige Belange zurückzustellen. Dieser „Disclaimer" soll vor allem einem Unmut darüber vorbeugen, dass im Folgenden vieles nicht angesprochen wird. Vor allem möchte das Nachfolgende den Versuch unternehmen, nach einer bereits intensiv geführten Debatte gleichwohl etwas zu „liefern", das neuen Diskussionsstoff bietet. Und natürlich konnten wir nicht dem Reiz widerstehen, dabei Bezüge zum aktuell laufenden Gesetzgebungsverfahren herzustellen, das sich der Triage widmet – zumal es hier um eine Regelung geht, die im rechtsvergleichenden Kontext einzigartig ist.

## II. Systematischer Teil

### *1. Rechtspositionen*

Die hier zu behandelnden Priorisierungen wirken sich auf höchstrangige Rechtsgüter bzw. grundrechtliche Positionen aus: Leben und körperliche Unversehrtheit bzw. Gesundheit sowie diverse Freiheitsrechte, deren aktuelle und künftige Ausübung sie beeinflussen. Sie sind mit Rechtspositionen verbunden, die ihre Erhaltung durch Verbote (ungerechtfertigter) Eingriffe, Sicherungs-, Schutz- und Hilfspflichten bzw. Teilhabeansprüche rechtlich „absichern" und jeder Person qua Geburt als Teil ihrer Rechtssphäre zuschreiben. Diese Rechtspositionen gehören zu einem System subjektiver, höchstpersönlicher Rechte. Fast

2) BVerfG, Beschl v. 16. 12. 2021 – 1 BvR 1541/20 –, Rdnr. 122.
3) BVerfG, Beschl. v. 16. 12. 2021 – 1 BvR 1541/20 –, Rdnr. 40.
4) Vgl. etwa § 11 Abs. 1 S. 1 Nr. 4 i. V. mit § 27 Abs. 1 S. 1 SGB V und Art. 20 Abs. 1 GG.
5) Art. 2 Abs. 2 S. 1 GG, s. a. *Brech*, Triage und Recht, 2008, S. 154 u. 336.
6) BVerfG, Beschl. v. 16. 12. 2021 – 1 BvR 1541/20 –, Rdnr. 5.
7) S. dazu etwa *Brech*, Triage und Recht, 2008, S. 151 u. 381 f.; s. a. *Brettel*, in: *Beckmann/Duttge/Gärditz/Hillgruber/Windhöfel* (Hrsg.), GS f. *Herbert Tröndle*, 2019.
8) BVerfG, Beschl. v. 16. 12. 2021 – 1 BvR 1541/20 –, Rdnr. 34.

alle Rechtsfragen, die diese Positionen betreffen, lassen sich mit diesem System lösen. Dabei werden subjektive Rechte zugeschrieben, Eingriffe grundsätzlich verboten, unter Umständen aber gerechtfertigt, und zwar entweder vom Betroffenen in eigener Kompetenz (Autonomie) oder nach allgemeinen Regeln, die letztlich den subjektiven Rechten Grenzen ziehen. Auch etliche Priorisierungsprobleme können so gelöst werden.

Doch nicht alle Priorisierungsprobleme lassen sich so lösen, und um die, bei denen das nicht möglich ist, geht es hier gerade. Sie sind durch folgende Situation gekennzeichnet: Zunächst besteht eine Knappheit von Ressourcen, derer es für den Erhalt der genannten Rechtsgüter bedarf. Zudem lässt sich kein Eingriff feststellen, der für die Zulässigkeit des weiteren Vorgehens relevant wäre (weil die Knappheit und die Unmöglichkeit, sie zu beheben, auf höherer Gewalt bzw. unwandelbaren Umständen beruhen, weil, soweit Dritte die Knappheit verursacht haben, diese von ihr nicht betroffen sind, bzw. weil, soweit Personen den Notstand beheben könnten, die Rechtsordnung sie nicht wirksam zu adressieren vermag). Und schließlich muss die Erfüllung rechtlich möglicher Hilfspflichten ebenfalls am Mangel verfügbarer Ressourcen scheitern (wiederum aus den gleichen Gründen).

Unter diesen Bedingungen stellt sich ein *echtes Verteilungsproblem*: Es muss wirklich eine neue, selbstständige Entscheidung über die Verteilung vorhandener Ressourcen getroffen werden, denn weder eine zuvor bereits vorgenommene, rechtlich verbindliche Zuordnung (subjektiver Rechte mit dem Verbot von ungerechtfertigten Eingriffen) noch eine Aktivierung weiterer Ressourcen durch Hilfspflichten etc. lösen das Problem. Insbesondere lässt sich das Problem also nicht durch die angeborenen Rechte und den Schutz der mit ihnen verbundenen Rechtssphären lösen. Das ist die unmittelbare Folge der angegebenen Voraussetzungen der Situation.

Gegenüber anderen Verteilungsproblemen zeichnen sich die medizinrechtlich interessierenden Fälle darüber hinaus dadurch aus, dass sie nicht mit den Mitteln des Wettbewerbs – d. h. nicht über Angebote derjenigen, die letztlich etwas erhalten möchten, und Präferenzentscheidungen anderer Personen, nicht über einen Markt – zu lösen sind und auch nicht über die Zuordnung von Bruchteilen. Zwar gilt es, die insgesamt verfügbaren Ressourcen aufzuteilen, aber eine Aufteilung, bei der letztlich jeder etwas erhält, und zwar einen in irgendeinem Sinne gleichen Anteil, kommt nicht in Betracht. Diese Konstellation des echten Verteilungsproblems sei im Folgenden als „echtes Priorisierungsproblem“ bezeichnet.

Oft wird bei einer solchen Problematik auf die begrenzten gegenständlichen Ressourcen geblickt: Impfstoff, Beatmungsmaschinen, passende Organe, Blutkonserven etc. in jeweils zu geringer Menge. Gefragt wird dann nach der Verteilung dieser Gegenstände. Prima facie kann das nahe liegen, denn dort besteht die „sichtbare“ Knappheit. In der Struktur des Problems ist diese Perspektive aber nicht vorgegeben. Das Problem besteht rechtlich im Notstand der angeborenen höchstpersönlichen Rechtsgüter (deren System löst das Problem nur nicht), und dieser Notstand lässt sich – zumindest in den medizinrechtlich relevanten Fällen – nicht unmittelbar durch die gegenständlichen Ressourcen beheben, sondern nur durch eine aktive Hilfeleistung (Behandlung) unter Verwendung der Ressourcen (Einspritzen des Impfstoffs, intensivmedizinische Behandlung unter Verwendung des Beatmungsgeräts etc.). Die Zuordnung der Mittel allein bessert die Lage der gefährdeten Rechtsgüter nicht, sondern nur ihr Einsatz im Rahmen einer insgesamt durchführbaren Behandlung. Isoliert über die Mittel zu entscheiden, wäre daher nicht sinnvoll, sondern würde höchstens eine weitere Verknappung bewirken. Der Unterschied ist keine reine Spitzfindigkeit, denn die Hilfeleistung erfordert unter Umständen etliche weitere Ressourcen, die ihrerseits knapp sein können, sie kann höchst unterschiedlich aufwendig sein, unterschiedliche Erfolge können zu erwarten sein, Erfolge können unterschiedlich wahrscheinlich sein, etc.

Beim echten Priorisierungsproblem ist daher darüber zu entscheiden, welchen hilfsbedürftigen Personen in welchem Umfang Hilfe zu leisten ist. Unterstellt wird dabei eine grundsätzliche Hilfspflicht (die ihren Grund letztlich in einer staatlichen Aufgabe der Für- und Vorsorge hat, im Detail aber hier nicht relevant ist) gegenüber allen im Notstand befangenen Personen. Die Verteilung bezieht sich daher auf die gesamte aktuell jeweils tatsächlich mögliche Hilfe. Nach den Voraussetzungen des echten Verteilungsproblems kann keine der betroffenen Personen einen unmittelbaren Anspruch auf die für sie nötige Hilfe oder die erforderlichen Mittel haben. Bestehen kann, wegen der Knappheit, nur ein Anspruch auf „angemessene“ Berücksichtigung bei der Verteilung.

### *2. Ex-post-Triage*

Wenn mit der Priorisierungsentscheidung zugunsten einer bestimmten Person (bzw. mehrerer Personen) ein dauerhafter Anspruch begründet würde, würden andere, insbesondere erst künftig im Notstand mitbefangene Personen, von der angemessenen Berücksichtigung ausgeschlossen, also in ihren Ansprüchen auf angemessene Berücksichtigung verletzt.

Die entgegenstehende Vorstellung, dass durch die Priorisierungsentscheidung eine solche dauerhafte Rechtsposition begründet würde, geht in die Irre: Sie ist der Versuch, doch eine Lösung innerhalb des Systems höchstpersönlicher Rechtspositionen zu erzwingen, zwar nicht unmittelbar mittels der angeborenen Rechte, aber doch zumindest mittels der durch Priorisierung zugeschriebenen Rechte. Auch dies passt aber nicht zur eben dargestellten Struktur des echten Priorisierungsproblems. Wenn neue Personen in die Notstandslage eintreten (oder sich die Lage im Notstand wesentlich ändert) ist nicht ersichtlich, weshalb die mitgebrachte Rechtsposition der einen besser sein soll als die der anderen, und das ist unabhängig davon, wann diese Rechtsposition begründet wurde[9]. Richtigerweise kann es schon deshalb kein generelles Verbot der „ex-post-Triage“ geben, denn das würde das verbleibende Residuum der Grundrechte Dritter auf Leben und körperliche Unversehrtheit in Form der angemessenen Berücksichtigung bei der Verteilungsentscheidung verletzen[10].

Die Vorstellung eines solchen Verbots ist regelmäßig damit verbunden, bestimmte Mittel würden einer bestimmten Person zugeordnet – quasi sachenrechtlich. Darum geht es aber eben nicht, sondern um Behandlung. Deshalb ist auch die strafrechtliche Vorstellung falsch, das „Entziehen“ des Mittels wäre eine Art Abbruch eines rettenden Kausalverlaufs oder „aktiver“ Behandlungsabbruch[11]. Die Mittel als solche retten niemanden, den vorausgesetzten Kausalverlauf gibt es nicht; er kann nicht abgebrochen werden. Es geht allein um eine Entscheidung über die Fortsetzung einer begonnenen Behandlung im Hinblick auf neuen oder geänderten Bedarf der im Notstand befangenen Personen.

9) Vgl. dazu auch *Hörnle*, in: *Hörnle/Huster/Poscher* (Hrsg.), Triage in der Pandemie, 2021, S. 149, 166 ff. Für ex-ante-Triage ebenso *Sowada,* NStZ 2020, 452, 456, danach aber ohne überzeugenden Grund anders für die ex-post-Triage (S. 456 ff.).

10) Vgl. auch *Gaede/Kubiciel/Saliger/Tsambikakis,* medstra 2020, 129, 134 ff.; *Gaede*, in: *Ulsenheimer/Gaede*, Arztstrafrecht in der Praxis, 6. Aufl. 2020, Rdnr. 1793.

11) Vgl. *Lindner*, MedR 2020, 723, 724 f. *Busch*, ZStW 132 (2020), 742, 775 ff.; *Deutscher Ethikrat*, MedR 2020, 468; *Jansen*, ZIS 2021, 15, 164 ff. Zur Kritik an dieser Einordnung auch *Hoven*, JZ 2020, 449, 452 ff.

Freilich besteht auch kein Gebot, permanent neu über die Verteilung zu entscheiden. Das Entscheiden selbst verbraucht Ressourcen, und das schränkt oft die Möglichkeiten der Hilfeleistung weiter ein. Schnelle Änderungen eines Behandlungsplans sind ebenfalls oft ineffizient[12]. Zu einer „angemessenen" Berücksichtigung aller Betroffenen trägt ineffizientes Vorgehen nicht bei. Eine Begrenzung der Häufigkeit von „ex-post-Triage" wird daher oft in ähnlichem Maße geboten sein, wie erst einmal die Wirkungen einer Behandlung abgewartet und eingeschätzt werden müssen, ehe über Änderungen nachzudenken ist. Aber für einen generellen oder weitgehenden Ausschluss der Möglichkeit, Priorisierungsentscheidungen zu revidieren bzw. im Lichte neuer Umstände neu zu treffen, besteht keine Grundlage.

### *3. Ausdrucksweisen*

Die Problematik ist, wie dargestellt, recht speziell. Das hat auch sprachliche Auswirkungen. Vielfach wird versucht, mittels bildhafter Sprache zu illustrieren oder mit Ausdrücken für anderweitig Bekanntes an Ähnlichkeiten anzuknüpfen. Das ist grundsätzlich sinnvoll, kann aber auch in die Irre führen. Daher sind einige Bemerkungen dazu angebracht.

Während der Corona-Pandemie wurde es üblich, das befürchtete Priorisierungsproblem auf Intensivstationen als „Triage" zu bezeichnen. Der Ausdruck wird unterschiedlich verwendet, insbesondere aber mit Bezug auf Krieg und verwundete Soldaten. Die Situation dort ist rechtlich speziell, weil sie durch eine Aufopferungssituation geprägt ist, die nicht nur den Grund des Notstands beeinflusst, sondern auch „Kampfkrafterhaltung" o.ä. als Entscheidungskriterium rechtfertigen kann. Im hier betrachteten echten Priorisierungsproblem geht es aber nicht um die Erreichung solcher externen (bzw. nur mittelbar und unspezifisch eigenen) Zwecke, sondern um eine Entscheidung im Rahmen der unmittelbaren eigenen Interessen der Betroffenen, und der Kern des Problems besteht darin, dass diese sich nur eingeschränkt verwirklichen lassen.

Man kann das Problem als Konkurrenz der Betroffenen darstellen, das aber weckt die unpassende Assoziation von Wettbewerb, um den es ja gerade nicht geht. Man kann es auch als Kollision ihrer Bedarfe auffassen. Dieses Bild wird hier vorgezogen, obwohl es insoweit schief ist, als nur Vorhandenes wirklich kollidieren kann, während es hier um das Fehlen von Ressourcen geht. Man kann das Bild stützen, indem man die Situation als einen Fall der Interessenkollision auffasst. In gewisser Weise kollidieren zugleich „Pflichten". Auch diese Ausdrucksweise ist aber schief: Pflichten müssen erfüllbar sein – nulla obligatio ultra posse. Soweit mehrere Pflichten nicht zugleich erfüllbar wären, können sie nicht gleichzeitig bestehen, also auch nicht kollidieren. Es bestehen vielmehr mehrere allgemeine Regeln (Gebote, Hilfe zu leisten), die in der konkreten Situation nicht zugleich zu erfüllen sind, einander in dieser Situation also widersprechen, und dieser Widerspruch ist durch die Priorisierungsentscheidung aufzuheben. Eine echte Pflicht besteht dann nur zur Umsetzung der Priorisierungsentscheidung. Streng genommen gibt es also keine „Pflichtenkollision", gleichwohl hat sich der Ausdruck für die genannte Problematik etabliert, und das schadet nicht, wenn man ihn in der angegebenen Weise richtig versteht (insbesondere als Ausschluss der Pflichtverletzung, also Rechtfertigungsgrund, und nicht erst Frage der Schuld[13]). Entsprechend ist es üblich, auch die kollidierenden Gebote als „Pflichten" zu bezeichnen; soweit das zu problematischen Mehrdeutigkeiten führt, kann man mit Ausdrücken wie „grundsätzliche" oder „generelle Pflicht" klarstellen, dass nur die allgemeine Regel und nicht die konkrete Pflicht gemeint ist.

In einer gleich noch zu klärenden Weise wird die Auswahl nach Kriterien zu erfolgen haben, die auch in Zielen bestehen können, die sich nur teilweise erfüllen lassen, aber möglichst weitgehend erfüllt werden sollen. Man kann sie daher als „Optimierung", das Gebot der Befolgung dieser Kriterien als „Optimierungsgebot" und die Auswahl unter Anwendung dieser Kriterien als „Abwägung" bezeichnen. Weitere, mit diesen Ausdrücken in anderen Zusammenhängen oft verbundene Implikationen, ergeben sich hier aber nicht. Insbesondere folgt aus einer solchen Sprechweise durchaus nicht, dass die Kriterien utilitaristisch sein sollten[14]. Es folgt auch keine Kompetenz bestimmter Personen oder des Staates zur Durchführung oder Regelung der „Abwägung".

### *4. Prozedurale Pflichten*

Regeln setzen in ihrem Tatbestand immer Umstände voraus, und deren Feststellung hat wiederum Regeln zu folgen. Das echte Priorisierungsproblem betrifft nicht nur die zentrale Regel, nach der die letztlich zu leistende Hilfe auszuwählen ist (Priorisierung 1. Ordnung), sondern auch viele potentiell relevante Umstände, die den Bedarf, Behandlungsmöglichkeiten, Behandlungsaussichten etc. näher kennzeichnen, und diese sind nur mit Schwierigkeiten festzustellen. Die Feststellungen verbrauchen selbst Ressourcen und limitieren die Hilfsmöglichkeiten weiter. Das führt dazu, dass auch bzgl. des Aufwands der einzelnen Feststellungen untereinander (Priorisierung 2. Ordnung) sowie zwischen der zu leistenden Hilfe und dem Aufwand für Feststellungen (ordnungsübergreifende Priorisierung) Priorisierungsentscheidungen zu treffen sind. Soweit die Auswahlkriterien nicht generell vorgegeben, sondern ihrerseits erst in der Situation festzustellen sind, z.B. weil diese selbst von den Betroffenen abhängen, gilt dafür das Gleiche wie für die Tatsachenfeststellungen.

Die Rechtsfrage, wie eine echte Priorisierung vorzunehmen ist, ist daher nicht einfach dadurch zu behandeln, dass man das Für und Wider verschiedener Auswahlkriterien erwägt und letztlich eine Priorisierungsregel vorschlägt. Vielmehr gibt es drei Arten von Regeln, die zu dieser Problematik gehören: 1. Regeln über die Feststellung der relevanten Kriterien (wenn diese nicht von vornherein feststehen), 2. Regeln über die Feststellung der relevanten Umstände und 3. Regeln über die Durchführung der Auswahl. Die zentrale Auswahlregel ist letztlich nur ein Teil der dritten Gruppe von Regeln. Im Rahmen der ersten und zweiten Gruppe ist darauf zu achten, dass es keineswegs einfach um eine bestmögliche Feststellung der Kriterien bzw. Umstände gehen darf, sondern dass die aufgestellten Anforderungen jeweils zu Lasten der Hilfsmöglichkeiten gehen. Über diese Regeln hinaus können und werden natürlich in früheren Phasen des Geschehens auch Pflichten zur Vermeidung der Kollision durch Prävention der Notstandslage[15] bestehen, aber sie lösen kein doch entstandenes Notstandsproblem und sind daher nicht Gegenstand der hier anzustellenden Überlegungen.

Die angegebenen Gruppen von Regeln sind im Wesentlichen prozeduraler Natur. Aus ihnen ergeben sich pro-

12) Insoweit zutreffend, wenn auch im Ergebnis zu weit gehend *Fateh-Moghadam/Gutmann*, in: *Hörnle/Huster/Poscher* (Hrsg.), Triage in der Pandemie, 2021, S. 291, 329ff.

13) Dazu auch *Gaede/Kubiciel/Saliger/Tsambikakis,* medstra 2020, 129, 132f.

14) Vgl. auch *Duttge*, medstra 2020, 193. Zu weiteren verbreiteten Fehlschlüssen und Fehlannahmen *Lübbe*, in: *Hörnle/Huster/Poscher* (Hrsg.), Triage in der Pandemie, 2021, S. 257ff.

15) Nötig ist dazu die Erhöhung der Ressourcen oder die Vermeidung des Bedarfs, nicht hingegen eine ihrerseits problematische präventive Triage, dazu *Gerson*, medstra 2021, 142ff.

zedurale Pflichten. Damit verschiebt sich die Perspektive von einem bloßen Maßstab, der angelegt werden soll, zum Vorgehen derjenigen, die priorisieren müssen. Auch das ist ein Fortschritt: Ein bloßer Maßstab führt bei Personen, die Hilfe leisten, eher zu Befürchtungen, ihn doch zu verfehlen, was dann haftungsrechtliche und strafrechtliche Konsequenzen haben kann. Gerade weil das Priorisierungsproblem vielschichtig (vgl. die eben dargestellten Ordnungen des Problems) und stark umstritten ist, liegt Furcht vor der nachträglichen Bewertung trotz redlichster Absichten auch durchaus nicht fern. Das Recht erfüllt seine Zwecke hier bislang nicht gut – es sollte einen Rahmen für das Handeln abstecken, aber eine viele Helfende ständig begleitende Angst zu erzeugen, ist unangemessen und dysfunktional. Mit Handreichungen für richtiges Vorgehen kann das Recht seine Aufgaben hier eventuell besser erfüllen.

### 5. *Selbstbestimmung*

Das medizinrechtliche echte Priorisierungsproblem hat eine weitere, bislang wenig beachtete Eigenheit: Die zentralen Belange, die im Notstand befangen sind, namentlich Leben und Gesundheit, unterliegen der Selbstbestimmung jedes Einzelnen für sich. Der sicherlich wichtigste Entwicklungsstrang des Medizinrechts der letzten Jahre und Jahrzehnte besteht in einer zunehmenden Ausrichtung auf die Selbstbestimmung in Form der Patientenautonomie. Ausdruck davon sind insbesondere die Entwicklung und Normierung der Patientenverfügung und des Behandlungsvertragsrechts sowie das jüngst vom BVerfG[16] ausgesprochene Recht auf selbstbestimmtes Sterben. Diese Autonomie und der Anspruch auf ihre Achtung werden vom BVerfG – sehr überzeugend – als wesentlicher Aspekt des in Art. 2 Abs. 1 i. V. mit Art. 1 Abs. 1 GG zu verortenden allgemeinen Persönlichkeitsrechts angesehen[17].

Es ist Sache jeder Person, für sich zu bestimmen, unter welchen Umständen sie leben möchte, was sie für sich als körperlich und geistig gesunden Zustand ansieht bzw. welchen Zustand sie anstrebt, welche Behandlungsmethoden sie vorzieht, welche Risiken sie eingehen möchte und auch, welche Prioritäten sie zwischen ihrem Leben, ihrer Gesundheit und anderen Interessen setzt. Solange sie – am Maßstab der Einwilligungsfähigkeit im Hinblick auf ihre höchstpersönlichen Rechtsgüter – die betreffenden Entscheidungen selbst zu treffen vermag, darf kein anderer – nicht der Staat, nicht die behandelnde Person, kein Dritter – ihre Entscheidungen durch eigene (oder bloßen Zufall[18]) ersetzen. Ein Anspruch auf Umsetzung durch andere Personen – etwa eine Pflicht der behandelnden Ärztin oder des Arztes, alle Behandlungswünsche zu erfüllen – besteht in der Regel freilich nicht, sondern auch für sie (in verschiedenen Ausprägungen) grundsätzlich Freiheit. Ein Anspruch auf Finanzierung bzw. Zurverfügungstellung der nötigen Mittel – insbesondere im Rahmen der gesetzlichen oder privaten Krankenversicherung – besteht ebenfalls keineswegs für alle eigenen Vorstellungen, sondern nur nach allgemeinen Kriterien. Im paradigmatischen Fall der Krankenbehandlung im Rahmen der gesetzlichen Krankenversicherung ist der Anspruch nach § 27 Abs. 1 SGB V zu bestimmen. Das geschieht zwar durch eine individuelle ärztliche Entscheidung (ggf. per Verordnung nach §§ 31 ff. SGB V), jedoch im Rahmen des Facharztstandards und im Hinblick auf die von den Kassenärztlichen Bundesvereinigungen mit dem Spitzenverband Bund der Krankenkassen getroffenen Vereinbarungen nach §§ 82 ff. SGB V. In beiderlei Rechtsrahmen manifestieren sich übliche Patientenpräferenzen, medizinisch-technische Möglichkeiten und Grenzen der Verfügbarkeit von Ressourcen. Auch bei dieser Bestimmung von Ansprüchen ist es aber nicht etwa Sache des Staates oder von Einzelpersonen, die Kriterien selbst festzulegen oder nach eigenen Präferenzen zu entscheiden[19].

Das echte Priorisierungsproblem ist eine durch besondere Ressourcenknappheit zugespitzte Variante der eben skizzierten Entscheidung über das Bestehen eines Anspruchs auf Behandlung. Anders als dort geht es bzgl. verfügbarer Ressourcen nicht nur um das Verhältnis zwischen einzelnen Versicherten und Interessen der Gemeinschaft (Wirtschaftlichkeit, § 12 SGB V), sondern vor allem um das Verhältnis der Interessen der Betroffenen. Für sich genommen konstituieren diese sich aber rechtlich richtigerweise immer auf dieselbe, durch die Selbstbestimmung der Betroffenen geprägte Weise.

Es liegt deshalb durchaus nicht nahe, das echte Priorisierungsproblem durch eine allgemeine gesetzliche Bestimmung von Auswahlkriterien behandeln zu wollen, auch wenn genau das immer wieder gefordert und politisch angestrebt wird[20]. Dabei hilft es auch nicht, wenn der Staat sich um weltanschauliche Neutralität bemüht. Im Ausgangspunkt ist es Sache der Betroffenen, ihr Priorisierungsproblem nach eigenen Präferenzen zu lösen.

### 6. *Mutmaßliche Einigung*

Dieser Befund liefert offenkundig nicht unmittelbar eine Lösung, denn tatsächliche Beratungen und eine tatsächliche Einigung der Betroffenen sind praktisch nicht vorstellbar. Gleichwohl ist der Befund nicht ohne Bedeutung. Zunächst lässt sich – ganz unabhängig von der Wahrscheinlichkeit bzw. Häufigkeit, dass das geschieht – der Fall denken, dass die Betroffenen sich auf Kriterien einigen. Dann wäre es ein Eingriff in das Selbstbestimmungsrecht der Beteiligten, wenn doch nach anderen Kriterien entschieden würde. Es lässt sich auch der Fall denken, dass die Betroffenen die von ihnen präferierten Kriterien vorab festgelegt haben (nach Art einer Patientenverfügung) und in ihren Festlegungen übereinstimmen – vor allem dann, wenn letztlich nur wenige Personen betroffen sind. Wiederum wäre es dann ein Eingriff in ihr Selbstbestimmungsrecht, wenn nach anderen Kriterien entschieden würde.

Das tatsächliche Unvermögen einer Person, in eigener Angelegenheit frei und selbstbestimmt zu entscheiden, hat früher zur weitgehenden Ersetzung ihrer Entscheidung durch eine fremde, d. h. eine Art der Bevormundung geführt. Würde man im Falle des Unvermögens der im Notstand befangenen Personen auf fremde, seien sie auch staatlich gesetzte Kriterien ausweichen, wäre das ein entsprechendes Vorgehen. Wie bei jeder Fremdgeschäftsführung ist richtigerweise aber als Maßstab der mutmaßliche Wille des oder der Betroffenen heranzuziehen, wenn ein wirklicher nicht gebildet wurde bzw. nicht gebildet werden kann (§ 677 BGB); im Betreuungsrecht gilt nichts anderes.

16) BVerfGE 153, 182, Rdnrn. 201 ff. Vgl. zu diesem Ausgangspunkt für Priorisierungsfragen auch *Taupitz*, MedR 2020, 440 f.

17) BVerfGE 153, 182, Rdnrn. 201 ff.

18) Dafür *Engländer*, in: *Hörnle/Huster/Poscher* (Hrsg.), Triage in der Pandemie, 2021, S. 111, 139 ff. und *Zimmermann*, Triage in der Pandemie, 2021, S. 221, 249 ff. Näher dazu bereits *Depenheuer*, JZ 1993, 171, 173 ff.

19) Auch das damit verbundene Problem der Abwägung von Leben gegen Leben, die jedenfalls die Würde der Betroffenen nicht verletzen darf – dazu *Poscher*, in: *Hörnle/Huster/Poscher* (Hrsg.), Triage in der Pandemie, 2021, S. 41, 43 ff., 65 ff. –, wird so vermieden.

20) Dazu etwa BT-Dr. 20/1483 Antwort auf schriftliche Frage Nr. 57; *Huster*, in: *Hörnle/Huster/Poscher* (Hrsg.), Triage in der Pandemie, 2021, S. 83, 90 ff., *Ehlers/Bartholomä/Menghin*, MedR 2021, 416, 417 ff.; *Streng-Baunemann*, ZIS 2021, 170, 175 ff. Zu Recht zurückhaltend *Hörnle*, in: *Hörnle/Huster/Poscher* (Hrsg.), Triage in der Pandemie, 2021, S. 149, 180 ff.

Mit Bezug auf das echte Priorisierungsproblem ist die Mutmaßung komplexer als mit Bezug auf eine Einzelperson. Geht es dort um die Frage, welchen Willen die Einzelperson in der aktuellen Situation bilden würde, wenn sie einen Willen bilden könnte, so geht es hier um die Frage, auf welche Kriterien die Betroffenen sich einigen würden, wenn sie sich einigen würden.

In beiden Fällen ist es kein durchgreifender Gegeneinwand, dass die jeweilige Willensbildung bzw. Einigung praktisch unmöglich ist. Dass sie es ist, ist der Grund, aus dem die Mutmaßung nötig wird. Es ist auch kein durchgreifender Einwand, dass selbst dann, wenn die Situation sich so verändern würde, dass die Person bzw. Gruppe prinzipiell eine eigene Entscheidung treffen könnte, sie sich eventuell doch zu keiner Entscheidung durchringen könnte. Gefragt wird nur, wie die Willensbildung bzw. Einigung wohl am ehesten aussähe, wenn sie zustande käme – dieser Erfolg wird zum Zwecke der Mutmaßung stets hypothetisch unterstellt[21]. Schließlich greift auch der Einwand nicht durch, dass nicht angegeben werden kann, auf welchem Wege eine Willensbildung bzw. Einigung hypothetisch herbeigeführt oder konstruiert werden sollte. Auch bei der ganz normalen eigenen Willensbildung und Einigung mündiger Personen lässt sich im Allgemeinen nicht angeben, wie der Wille bzw. die Einigung genau zustande kommt. Ein Verfahren zur gezielten Konstruktion der Mutmaßung gibt es in beiden Fällen nicht. Trotzdem lässt sich mit der Figur praktisch arbeiten.

### *7. Spielräume und Vorgaben*

Zunächst ist von bekannten Präferenzen der Betroffenen auszugehen. Zur Bestimmung des mutmaßlichen Willens einer Person ist das entscheidend. Für die mutmaßliche Einigung weniger Personen ist das ähnlich. Je mehr Personen im Notstand befangen sind, desto weniger relevant und zugleich aufwändiger würde diese Feststellung jedoch. Je größer die Zahl der Betroffenen ist, je stärker die Zusammensetzung ihrer Gruppe derjenigen der Allgemeinbevölkerung entspricht, und je länger sich der Notstand fortsetzt, desto weniger ist davon auszugehen, dass eine Einigung von individuellen Präferenzen geprägt würde. Zugleich wird es dann immer plausibler anzunehmen, dass eine Einigung innerhalb der Gruppe das gleiche Ergebnis hätte wie eine innerhalb des repräsentativen Gesetzgebungsorgans. Für die Priorität bei Organtransplantationen ist gegen eine gesetzliche Regelung des Verfahrens, der generellen Kriterien und der Kompetenz zur Konkretisierung, wie sie tatsächlich besteht (§ 12 Abs. 3 S. 1 TPG zusammen mit insb. §§ 10, 12, 16 ff. TPG), daher nichts einzuwenden (zu weiteren Fällen III.2). Je kleiner und homogener aber die Gruppe der Betroffenen ist, und je zeitlich begrenzter der jeweilige Notstand, desto weniger plausibel ist eine solche Annahme.

Die Perspektive der mutmaßlichen Einigung ist notwendig mit Einschätzungsspielräumen derjenigen, die die Mutmaßung anzustellen haben, verbunden. Rechtlich fehlerhaft ist deren Vorgehen nur in besonderen Fällen, nämlich dann, wenn sie zuvor normierte konkrete prozedurale Pflichten verletzen, eigene Präferenzen an die Stelle von Annahmen über Präferenzen der Betroffenen setzen oder von fremdvorgegebenen Kriterien ausgehen. Wer etwa als Rassist selbst rassistisch entscheidet oder allgemeine Maßgeblichkeit utilitaristischer Kriterien unterstellt, handelt rechtsfehlerhaft. Soweit hingegen eine – mit Blick auf andere dringliche Tätigkeiten eventuell auch nur oberflächliche – Befassung damit, welche Präferenzen der Betroffenen plausibel anzunehmen sind, und darauf aufbauend eine Mutmaßung erfolgt, ist das nicht zu beanstanden.

Die Perspektive der mutmaßlichen Einigung hat eine weitere Konsequenz: Die herangezogenen Kriterien müssen nicht weltanschaulich neutral sein. Der Staat ist bei eigenem Handeln und eigener Regelung zu weltanschaulicher Neutralität verpflichtet[22]. Die Betroffenen sind das aber gerade nicht. Auch für Mutmaßungen über ihre Präferenzen, Willen bzw. Einigung besteht eine solche Bindung nicht, denn gerade die Selbstbestimmung und damit auch Weltanschauung der Betroffenen soll ja respektiert werden[23]. Wiederum gilt: Je größer die Zahl der Betroffenen ist und je heterogener ihre Gruppe zusammengesetzt ist, desto plausibler ist die Annahme, dass eine Einigung nur auf weltanschaulich neutrale Kriterien erfolgen würde. Je kleiner und homogener die Gruppe aber ist, desto weniger ist das der Fall.

Geregelt werden können freilich stets prozedurale Pflichten, wie sie oben dargestellt wurden. Für die Durchführung der Auswahl kann auch geregelt werden, welche Präferenzen zu vermuten sind, wenn keine anderen Informationen vorliegen. Treffen kann solche Regelungen der Gesetzgeber. Oft wird es indes durchaus naheliegen – wie schon im Beispiel des TPG –, an innermedizinische Verfahren, Richtlinien, Empfehlungen etc. anzuknüpfen, denn diejenigen, die tagtäglich mit Patienten in vergleichbaren Situationen umgehen, werden in der Regel die besten Vorstellungen von deren Präferenzen haben[24]. Die Regelungsaufgabe besteht aber eben nicht darin, eine allgemeine Vorrangregel zur Lösung der Kollision innerhalb des Systems angeborener höchstpersönlicher Rechtsgüter aufzustellen. Vielmehr geht es um die Wahrung der mit dem Selbstbestimmungsrecht der Einzelnen verbundenen Kompetenzen. Jeder Eingriff in dieses, auch durch eine allgemein vorgegebene Vorrangregel, bedürfte einer gesonderten verfassungsrechtlichen Rechtfertigung.

## III. Umsetzung (das praktische Wie)

### *1. Priorisierungskriterien*

Um diese Überlegungen nun noch etwas weiterzuführen, sollen – wie angekündigt (s. Abschnitt I.) – die Kriterien für Priorisierungsentscheidungen aus einer anderen Perspektive in den Blick genommen werden. Über diese Kriterien wurde und wird lebhaft diskutiert, was schon deshalb nachvollziehbar ist, weil es vor allem auf sie sowohl im jetzt laufenden Gesetzgebungsverfahren als auch in der Praxis ankommt. Die Liste der dabei erwogenen Kriterien ist lang: Medizinrechtliche Hauptaspekte wie medizinische Indikation, Patientenwillen[25] oder Patientenwohl[26] gehören ebenso dazu wie klinische Erfolgsaussichten[27], die Dringlichkeit, medizinische Bedürftigkeit, der erwartbare medizinischer Nutzen, die Kosteneffektivität[28], der Allgemeinzustand[29] oder Komorbiditäten als „spezifisch fehlende physiologische Ressourcen“[30].

21) Insofern unterscheiden sich die hier anzustellenden Überlegungen von denen von *John Rawls* (und von den darauf aufbauenden von *Zimmermann*, in: *Hörnle/Huster/Poscher* (Hrsg.), Triage in der Pandemie, 2021, S. 221 ff.).

22) Eingehend *Augsberg*, in: *Hörnle/Huster/Poscher* (Hrsg.), Triage in der Pandemie, 2021, S. 3, 10.

23) Empirisch zu den Unterschieden *Hoven*, in: *Hörnle/Huster/Poscher* (Hrsg.), Triage in der Pandemie, 2021, S. 335, 336 ff.

24) So sind die klinisch-ethischen Empfehlungen der DIVI über die Entscheidungen über die Zuteilung intensivmedizinischer Ressourcen im Kontext der COVID-19-Pandemie (2. Fassung v. 17. 4. 2020) dort, wo sie eine eigene Haltung darlegen und nicht über rechtliche Risiken spekulieren, sehr gelungen.

25) BVerfG, Beschl. v. 16. 12. 2021 – 1 BvR 1541/20 –, Rdnr. 32.

26) BVerfG, Beschl. v. 16. 12. 2021 – 1 BvR 1541/20 –, Rdnr. 35.

27) BVerfG, Beschl. v. 16. 12. 2021 – 1 BvR 1541/20 –, Rdnr. 10.

28) BVerfG, Beschl. v. 16. 12. 2021 – 1 BvR 1541/20 –, Rdnr. 37.

29) BVerfG, Beschl. v. 16. 12. 2021 – 1 BvR 1541/20 –, Rdnr. 9.

30) BVerfG, Beschl. v. 16. 12. 2021 – 1 BvR 1541/20 –, Rdnr. 38.

*2. Mutmaßlich kollektiver Wille*

Was dazu nun im Folgenden entwickelt werden soll, setzt an das schon angesprochene Zusammenspiel von Tatsachen- und Rechtsfragen (s. Abschnitt II.4) mit der Vorstellung an, dass Erkenntnisregeln zu weiteren Konkretisierungen bei der Frage nach der Herleitung von Priorisierungskriterien verhelfen. So wurde im vorangehenden Abschnitt das allgemeine Persönlichkeitsrecht nach Art. 2 i. V. m. Art. 1 GG als zentraler „Hintergrundwert" identifiziert, an dem auch die Herleitung von Kriterien für Priorisierungsentscheidungen auszurichten sind. Dabei geht es um die jeweils selbstbestimmte Ausgestaltung der persönlichen Belange. Welche individuellen Lebensentwürfe im Zeitpunkt der Priorisierungsentscheidung allerdings aktuell sind, lässt sich nicht feststellen. Unerreichbar ist auch der ideale Prozess einer Einigung in der politischen Gemeinschaft, der im vorherigen Abschnitt skizziert wurde. Diese Gemeinschaft kann weder zu den konkret anstehenden Priorisierungsentscheidungen noch zu den zugrunde liegenden Wertentscheidungen befragt werden. Der Erkenntnisprozess trifft hier also auf die Grenze der Aufklärbarkeit im Tatsächlichen. Damit müssen die geforderten Entscheidungen auf anderem basieren als auf einer – eigentlich vorzugswürdigen – Tatsachenkenntnis. Zugleich gibt es für diesen Ersatz der Tatsachenkenntnis etablierte Orientierungshilfen (s. o.), etwa den Rückgriff auf Vermutungen bzw. Mutmaßungen oder auf Erfahrungsregeln.

Weil es nun im vorliegenden Betrachtungszusammenhang um allgemeinverbindliche Kriterien geht, müssen die Mutmaßungen dazu auch für die politische Gemeinschaft im Ganzen und nicht nur für den Einzelnen Geltung beanspruchen können. Es kann also nur um Kriterien gehen, die von der politischen Gemeinschaft im Falle einer Befragung mutmaßlich aufgestellt würden, auf die sich somit die Gemeinschaft in Ausübung ihrer kollektiven Autonomie einigen würde. Dabei sollte – wie bei der herkömmlichen mutmaßlichen Einwilligung – natürlich die Wahrscheinlichkeit einer Invalidität der jeweiligen Mutmaßung und damit des jeweiligen Kriteriums möglichst gering sein.

Was dies nun für die Herleitung von Auswahlkriterien bedeutet, lässt sich beispielhaft unter Bezug auf das Priorisierungskriterium der klinischen Erfolgsaussicht veranschaulichen: Damit kann zum Beispiel die Aussicht auf ein selbstbestimmtes Leben gemeint sein, andererseits aber auch – allein – die Wahrscheinlichkeit, die aktuelle Erkrankung zu überleben[31]. Dass sich die politische Gemeinschaft auf diese zweite Variante im idealen Prozess kollektiver Willensbildung einigen kann, liegt wesentlich näher als ein Konsens im Hinblick auf Prioritäten für das weitere Leben. Aus dem Blickwinkel der Validität erscheint somit die akute Überlebensaussicht gegenüber anderen Bezugsmomenten für die Erfolgsaussicht vorzugswürdig, was natürlich andere Begründungen für einen Vorrang nicht ausschließt. Mit dem mutmaßlichen Willen der politischen Gemeinschaft ist jedenfalls auch begründbar, dass es allein um die Erfolgsaussicht im Sinne einer Wahrscheinlichkeit gehen dürfe, die aktuelle Erkrankung zu überleben[32]. Dies betont auch das BVerfG. Maßgeblich könne allenfalls das Überleben im „jetzt und hier"[33] sein, also die Bewertung der kurzfristigen Überlebensaussicht, nicht der längerfristigen Lebensaussichten oder gar des Lebens selbst.

*3. Zurückhaltung mit Tatsachenbezügen*

Auch in anderer Hinsicht sind Erkenntnisregeln an der Grenze der Aufklärbarkeit im Tatsächlichen von Bedeutung, wenn es um die Herleitung von Kriterien für die Priorisierung bei Ressourcenknappheit geht: Natürlich muss die Auswahlentscheidung bestmöglichen Realitätsbezug haben, weshalb beispielsweise – so das BVerfG – die Sachgesetzlichkeiten der klinischen Praxis zu beachten sind[34]. Nun können Gesetze nur in langwierigen Prozeduren abgeändert werden und deshalb mit dem rasanten medizinischen Fortschritt nicht mithalten. Ein weiteres Problem bei Kriterien mit Tatsachenbezug – wie etwa der Überlebensaussicht – ist die allgemeine Verbindlichkeit. Denn die Vielgestaltigkeit der Realität hindert in großem Umfang an der Benennung von ausnahmslos gültigen Parametern. Allgemein, das heißt fallübergreifend verbindlich, lässt sich zumeist nur wenig sagen. Wenn Verallgemeinerungen aber mit einem relevanten „Wirklichkeitsverlust" einhergehen, dann bringen sie zwangsläufig Kriterien hervor, die eine tatsächlich bestehende Diversität nicht in der gebotenen Weise abbilden.

Dieser Aspekt steht auch im Zentrum der Entscheidung des BVerfG zur Triage. Dem Gericht ging es vorrangig um Vorkehrungen zur Vermeidung von tatsachenbezogenen Fehleinschätzungen. Den Gesetzgeber forderte das BVerfG vor allem dazu auf, der Gefahr entgegenzutreten, dass die Lage von Menschen mit Behinderungen falsch eingeschätzt wird. Für diese Menschen bestehe etwa die Gefahr der Benachteiligung, weil Beeinträchtigungen durch Behinderungen als Komorbiditäten oder Gebrechlichkeit gesehen würden[35]. Beim Abstellen auf die klinische Erfolgsaussicht hätten Menschen mit Behinderung ein durchweg höheres Risiko schlechterer Einstufung[36]. Wegen ihrer Abhängigkeit von Assistenz würden sie beispielsweise für gebrechlich gehalten[37]. Auch Messskalen würden ihnen negative Aussichten zuschreiben[38] – nicht zuletzt weil nicht behinderte Menschen unhinterfragt zum Maßstab genommen würden[39]. Skalen und Scores bezögen sich auf durchschnittliche Menschen und seien nicht für Menschen mit Behinderungen angeglichen worden[40]. Auch würden beispielsweise unbewusste Denkmuster beim Krankenhauspersonal zu Vorurteilen und Fehlprognosen führen[41]. Kurzum: Stereotypisierungen, Schemata und Pauschalisierungen würden behinderte Menschen bei medizinischen Entscheidungen in Orientierung an der klinischen Erfolgsaussicht benachteiligen[42].

Nun lässt sich (auch) in diesem Zusammenhang nicht grundsätzlich ändern, dass es an Tatsachenwissen fehlt. Die relevanten Tatsachen sind zum Teil schlicht nicht bestimmbar, weshalb an die Stelle von Feststellungen allenfalls Vermutungen und Einschätzungen treten können. Dass allerdings auch dabei (prozedurale) Pflichten gelten, ist bereits angeklungen (s. Abschnitt II.4); Erkenntnisregeln für die Feststellung von Tatsachen – wie etwa Konsistenz und Transparenz – sind selbstverständlicher Bestandteil des Rechts.

Dabei erscheint auch hier denkbar, Mutmaßungen an die Stelle von Tatsachen zu setzen, was Ergebnis einer normativen Entscheidung wäre, die von Erkenntnisregeln gesteuert sein kann. Soweit allerdings von jenen, die für die Regelsetzung zuständig sind, die Feststellung von Tatsachen gefordert wird, kommt eine Mutmaßung nicht in Betracht. Soweit sich die politische Gemeinschaft also beispielsweise dafür entscheidet, dass allein festgestellte Tatsachen einer Priorisierungsentscheidung zugrunde gelegt werden dürfen, darf nicht stattdessen auf Mutmaßungen dazu abgestellt werden. Wenn also zum Beispiel die Überlebenswahrscheinlichkeit als Priorisierungskriterium maßgeblich sein soll, kann als Ersatz dafür keine fingierte Überlebenswahr-

31) BVerfG, Beschl. v. 16. 12. 2021 – 1 BvR 1541/20 –, Rdnr. 120.
32) BVerfG, Beschl. v. 16. 12. 2021 – 1 BvR 1541/20 –, Rdnrn. 116 und 120.
33) BVerfG, Beschl. v. 16. 12. 2021 – 1 BvR 1541/20 –, Rdnr. 121.
34) BVerfG, Beschl. v. 16. 12. 2021 – 1 BvR 1541/20 –, Rdnr. 127.
35) BVerfG, Beschl. v. 16. 12. 2021 – 1 BvR 1541/20 –, Rdnr. 22.
36) BVerfG, Beschl. v. 16. 12. 2021 – 1 BvR 1541/20 –, Rdnr. 48.
37) BVerfG, Beschl. v. 16. 12. 2021 – 1 BvR 1541/20 –, Rdnr. 61.
38) BVerfG, Beschl. v. 16. 12. 2021 – 1 BvR 1541/20 –, Rdnr. 61.
39) BVerfG, Beschl. v. 16. 12. 2021 – 1 BvR 1541/20 –, Rdnr. 51.
40) BVerfG, Beschl. v. 16. 12. 2021 – 1 BvR 1541/20 –, Rdnr. 51.
41) BVerfG, Beschl. v. 16. 12. 2021 – 1 BvR 1541/20 –, Rdnr. 51.
42) BVerfG, Beschl. v. 16. 12. 2021 – 1 BvR 1541/20 –, Rdnr. 113.

scheinlichkeit dienen. Dies schließt keine Mutmaßungen dazu aus, ob es nach dem Willen der Rechtsgemeinschaft auf die Überlebenswahrscheinlichkeit ankommen soll. Denn Mutmaßungen sind stets ein Ersatz, wenn Feststellungen nicht getroffen werden können, aber ein Surrogat für diese Feststellungen für nötig gehalten wird.

Dabei besteht insbesondere dann die Notwendigkeit, an Stelle von festgestellten Tatsachen auf Mutmaßungen zurückzugreifen, wenn eine Entscheidung mit Bezug auf die Wirklichkeit getroffen werden *muss*. Und diese Konstellation liegt auch im Hinblick auf Maßstäbe für eine Priorisierungsentscheidung vor. Denn im Fall der Triage haben die Beteiligten nicht die Möglichkeit, sich aus den Priorisierungen „herauszuhalten". Denn Priorisieren heißt, etwas vorrangig zu behandeln, zu bevorzugen. In den Situationen der Triage sind Rettungschancen ohne Zutun der Retter aber nicht realisierbar und Retter zugleich rechtlich verpflichtet, aktiv zu werden[43]. Wenn sie dann ihrer Pflicht zum Einschreiten im Dienst der Realisierung von Rettungschancen nachkommen (also zum Beispiel das letzte der verfügbaren Beatmungsgeräte anschließen), vernichten sie zwangsläufig bei Kapazitätsmängeln die Rettungschancen von denen, für die die Ressourcen dann nicht mehr ausreichen.

Dabei können Retter ihr Eingreifen auf das beschränken, was zur Realisierung der ihnen am nächsten stehenden Rettungschance notwendig ist. Im Hinblick auf die Beeinflussung der Ressourcenverteilung betreiben sie dann minimalen Aufwand, indem sie beispielsweise keinen Abgleich mit Priorisierungskriterien vornehmen. Retter können aber auch weitergehend Einfluss auf die Ressourcenverteilung nehmen und Prinzipien anwenden, nach denen verteilt wird. In beiden Fällen treffen sie eine Wertentscheidung, also insbesondere auch dann, wenn sie auf zusätzlichen Aufwand für eine Beeinflussung der Ressourcenverteilung verzichten. Nicht zu ihren Handlungsmöglichkeiten gehört die fehlende Positionierung zur Ressourcenverteilung – sozusagen ein „Sich-Heraushalten". Ihnen bleibt nicht die Möglichkeit, keine Rolle in den Priorisierungsabläufen zu spielen, an der Priorisierung also nicht beteiligt zu sein. Für irgendetwas müssen sie sich entscheiden – und sei es fürs Nichtstun. Wenn sie gleichzeitig aber die relevanten Tatsachen für diese notwendigen Entscheidungen nicht feststellen können, dann bleiben ihnen nur Mutmaßungen. Hier müssen Mängel des Tatsachenwissens also mithilfe von Mutmaßungen überwunden werden. Hingegen können Priorisierungskriterien mit Tatsachenbezug jederzeit aufgegeben werden, wenn sie unbrauchbar sind.

Auch bei der Priorisierung im Fall der Ressourcenknappheit ist deshalb unseres Erachtens Zurückhaltung mit tatsachenbezogenen Auswahlkriterien geboten. Die Fehleranfälligkeit von Einschätzungen auf dieser Grundlage – etwa mit Bezug auf die Erfolgsaussicht – ist in einem Mittelfeld so groß, dass naheliegt, auf solche Kriterien allenfalls in Extremfällen an den äußeren Rändern des Möglichkeitsraums abzustellen. Gewissheiten kann es zum Beispiel im Hinblick auf das (zukunftsgewandte) Kriterium der Erfolgsaussicht ohnehin nicht geben. Für Vorsicht beim Rückgriff auf tatsachenbezogene Vorgaben spricht im Übrigen auch das Erfordernis der Handhabbarkeit bzw. Praktikabilität von Kriterien.

Das legt insgesamt Zurückhaltung beim Abstellen auf empirische Indikatoren nahe. Damit wollen wir nicht die Notwendigkeit einer gesetzlichen Regelung zur Auswahlentscheidung in der Akutsituation relativieren. Zur Prävention einer Triage wurden zahlreiche Verordnungen und Gesetze in Kraft gesetzt. Für die Auswahlentscheidung in der Akutsituation hingegen fehlt momentan ein national oder international konsentiertes System, es gibt keine allgemein geltenden oder rechtlich verbindlichen Standards[44]. Dabei stellt sich die Frage nach ihrer Einführung inzwischen in Deutschland nicht mehr. Denn das BVerfG hat die „unverzügliche" Schaffung eines entsprechenden Gesetzes gefordert und dabei darauf hingewiesen, dass der Gesetzgeber schon 2012 im Rahmen einer Risikoanalyse im Bevölkerungsschutz zur Einführung entsprechender gesetzlicher Regelungen aufgefordert wurde[45].

## Fazit

Unser Fazit lautet somit, dass der Schwerpunkt von Vorgaben bei prozeduralen Pflichten und nicht bei allgemeinverbindlichen Auswahlkriterien liegen sollte. Auch dies lässt eine Entlastung vom Verantwortungsdruck bei den Entscheidern vor Ort erwarten. Gerade prozedurale Vorgaben können die Sicherheit vermitteln, mit einer Berücksichtigung von wenigen Umständen das Richtige zu tun.

Mit prozeduralen Regeln meinen wir dabei moderate Vorgaben zur Erkenntnisgewinnung (also Erkenntnisregeln) oder zu den Zuständigkeiten. Dabei sollte auf keinen Fall der Aufwand aus dem Blick geraten, der mit jeder Vorgabe verbunden ist. Auch hier möchten wir deshalb an den Gesetzgeber appellieren, dass er behutsam bleibt. Mit jedem Dokumentationserfordernis und jeder prozeduralen Vorgabe wie einem Vier-Augen-Prinzip steigt die Belastung der Ressourcen, die dann für die Rettung selbst ggf. nicht zur Verfügung stehen.

Damit liegen unseres Erachtens auch Folgerungen im Hinblick auf die Strafbarkeitsrisiken nahe: Die Kriminalisierung eines Verhaltens setzt einen klaren gesellschaftlichen Konsens im Hinblick auf eine hinreichende bestimmte Verhaltensnorm voraus. Solange es keine allgemeinverbindlichen Verhaltenskriterien gibt, kommt eine strafrechtliche Haftung als Ergebnis einer Verhaltensbewertung nicht in Betracht. Wenn es ex ante keine klare Priorität gibt, dann fehlt es an einer Verhaltensnorm für die Priorisierungsentscheidung, die strafrechtlich abgesichert werden könnte.

Auch ist schon angeklungen, dass in den vieldiskutierten Fällen der Triage eine Rechtsverpflichtung besteht, über die vorhandenen Rettungsmöglichkeiten zu entscheiden[46]. Die Entscheider vor Ort müssen also – anders als beispielsweise der Kampfpilot, der ein entführtes Flugzeug eskortiert – aktiv den Lauf der Dinge beeinflussen[47]. Und gleichzeitig fehlt die Alternative eines Verhaltens, das nicht zu tödlichen Verläufen führt. Menschen in dieser Konstellation nicht zu retten, ist als kategorial andere Unrechtsform anzusehen, als sie zu töten. Und eine Einstufung als Unrecht setzt voraus, dass es eindeutig bessere Verhaltensoptionen gibt, was in den betrachteten Fällen offensichtlich nicht der Fall ist[48].

**Open Access.** Dieser Artikel wird unter der Creative Commons Namensnennung 4.0 International Lizenz veröffentlicht, welche die Nutzung, Vervielfältigung, Bearbeitung, Verbreitung und Wiedergabe in jeglichem Medium und Format erlaubt, sofern Sie den/die ursprünglichen Autor(en) und die Quelle ordnungsgemäß nennen, einen Link zur Creative Commons Lizenz beifügen und angeben, ob Änderungen vorgenommen wurden.
Die in diesem Artikel enthaltenen Bilder und sonstiges Drittmaterial unterliegen ebenfalls der genannten Creative Commons Lizenz, sofern sich aus der Abbildungslegende nichts anderes ergibt. Sofern das betreffende Material nicht unter der genannten Creative Commons Lizenz steht und die betreffende Handlung nicht nach gesetzlichen Vorschriften erlaubt ist, ist für die oben aufgeführten Weiterverwendungen des Materials die Einwilligung des jeweiligen Rechteinhabers einzuholen. Weitere Details zur Lizenz entnehmen Sie bitte der Lizenzinformation auf http://creativecommons.org/licenses/by/4.0/deed.de.

43) S. dazu *Brettel*, in: GS f. *Tröndle*, 2019, S. 415.
44) BVerfG, Beschl. v. 16.12.2021 – 1 BvR 1541/20 –, Rdnr. 7.
45) BVerfG, Beschl. v. 16.12.2021 – 1 BvR 1541/20 –, Rdnr. 45.
46) *Brettel*, in: GS f. *Tröndle*, 2019, S. 415.
47) *Brettel*, in: GS f. *Tröndle*, 2019, S. 415.
48) *Brettel*, in: GS f. *Tröndle*, 2019, S. 412.

Funding

Open access funding enabled and organized by Projekt DEAL.

